# Quality of life outcomes following penile prosthesis insertion in the post-ischaemic priapism setting

**DOI:** 10.1038/s41443-025-01073-y

**Published:** 2025-07-25

**Authors:** Isabel Dighero, Katy Naylor, Aisling Looney, Fiona Holden, Chloe Mount, Mark Johnson, Wai Gin Lee, David Ralph, Philippa Sangster

**Affiliations:** 1 University College London Hospital (UCLH), London, UK; 2 University College London (UCL), London, UK; 3 St Vincent’s University Hospital, Dublin, Ireland; 4 Buckinghamshire Healthcare NHS Trust, High Wycombe, UK; 5 Leeds Teaching Hospitals NHS Trust, Leeds, UK; 6 St Peter’s Andrology, London, UK

**Keywords:** Erectile dysfunction, Sexual dysfunction

## Abstract

Penile prosthesis insertion is recommended for long-duration ischaemic priapism patients with refractory erectile dysfunction. There is a paucity of published data focusing on long term outcomes and quality of life reporting for patients in this setting. We contacted patients who had a post-ischaemic priapism penile prosthesis inserted in our department via telephone and conducted the previously validated Quality of Life and Sexuality with Penile Prosthesis (QoLSPP) questionnaire. Question items were answered on a Likert scale from 0–5 with satisfactory scores ≥3. Two-tailed Z-tests were used to determine satisfactory scores at significant levels (*p* < 0.05). We chose to add two additional questions on regret and feelings towards living the rest of their lives with a penile prosthesis. 167 patients had penile prostheses inserted post- ischaemic priapism between 2002–2022. Of these, 39 (23.4%; implants between 2007–2021) completed our questionnaire. The median age was 56 years (IQR 50–63) with a median time to questionnaire post-ischaemic priapism of 9 years (IQR 3–11). Analysing the QoLSPP mean item responses, the penile prosthesis resulted in satisfactory scores in 7 of 16 QoLSPP questionnaire items: device adequacy, device rapidity, device duration, meeting expectations, contentment with life, general well-being and sexual experience. The pooled mean score by domain was 3.7 ± 1.2 for functional, 3.3 ± 1.4 for relational, 3.2 ± 1 for social and 3.4 ± 1.1 for personal. Sub-group analysis demonstrated no significant difference in mean score by prosthesis type (inflatable versus malleable). Ten respondents cited specific reasons for how the penile prosthesis caused dissatisfaction. All 39 respondents (100%) answered ‘yes’, they did not regret penile prosthesis insertion as a treatment option and that they would be satisfied living the remainder of their lives with a device in situ. This study can be used to inform patient counselling, that penile prosthesis insertion is a suitable surgical technique to maintain sexual function post-ischaemic priapism.

## Introduction

Priapism is defined as penile erection that persists beyond 4 hours and is unrelated to sexual interest or stimulation [1]. The incidence of priapism is rare (0.5–0.9 cases per 100,000 person-years). Ischaemic priapism is the most common form, accounting for an estimated 95% of all priapism episodes [2]. Ischaemic priapism is characterised by intracavernosal pressures that compromise circulation. Without emergency intervention, ischaemic priapism risks irreversible corporal fibrosis which can lead to refractory erectile dysfunction (ED). Initial management mandates a trial of conservative measures such as cavernosal aspiration, irrigation and intra-cavernosal therapies, followed by emergency shunt surgery to achieve detumescence. Placement of a penile prosthesis (PP) is a well-established therapeutic option that should be considered for delayed or refractory ischaemic priapism, leading to refractory ED, and is recommended in international guidelines [1, 3, 4]. Optimal time for implantation is with in the first three weeks of a priapism episode, however, significant bruising or history of a shunt may delay the definitive surgery. The two most commonly utilised subtypes of PP are the inflatable penile prosthesis (IPP) and the malleable penile prosthesis (MPP) [5].

Insertion of PP in the post-ischaemic priapism setting is uniquely challenging. Structural changes occur due to cavernosal tissue necrosis and fibrosis with consequent penile scarring, deformity and shortening [6, 7]. There are therefore additional challenges in achieving full satisfaction with PP amongst post-ischaemic priapism patients. In severe cases, additional surgical techniques may have been be required at the time of PP insertion such as corporal excavation, excision of scar tissue, use of small-diameter prosthesis and even penile reconstruction [8–10]. All these caveats may lead to increased post operative complications and significant penile shortening. How this affects the lifetime experience of PP for these patients remains to be fully understood.

To date, no study has reported findings dealing with quality of life (QoL) outcomes selectively amongst the post-ischaemic priapism PP patient cohort. This is understandable given the rarity of priapism. A comprehensive multicentre registry illustrates that the most common indications for undergoing PP insertion are refractory ED due to post radical prostatectomy (28%), diabetes (21.6%), cardiovascular disease (19.6%), or Peyronie’s disease (8.9%). In fact, just 19 (1.4%) of this 1348 patient cohort had PP insertion post-ischaemic priapism [11]. The authors report that 16 (84.2%) of the 19 patients who underwent PP post-ischaemic priapism were ‘satisfied’ or ‘very satisfied’ with their device at 2 years after surgery. Several other studies have reported on satisfaction post PP for priapism. Reported satisfaction rates range from 80–100% for IPP and 60.5–100% for MPP, with sexual intercourse achievement ranging between 64.2–100% [9, 12–16]. However, studies have so far lacked a standardised approach to assessing satisfaction. There is therefore a need for research which adds to this understanding and represents a more comprehensive assessment of patients’ outcomes utilizing a validated questionnaire.

In 2014, Caraceni and Utizi proposed the Italian-language Quality of Life and Sexuality with Penile Prosthesis (QoLSPP) questionnaire as a validated method of evaluating QoL post-PP insertion [17]. This questionnaire was developed to provide a reliable method of assessing both perceived PP function and postoperative QoL. QoLSPP was validated by the authors in a cohort of 69 patients treated with IPP for ED. Several Italian studies have replicated findings demonstrating high satisfaction rates with PP in cohorts of ED patients using the QoLSPP [18–20]. Carlos et al. were the first to apply an English language version of QoLSPP amongst a cohort of 90 patients with PP [21]. These authors mirror results reported from Italy and demonstrate high prosthesis-specific sexual QoL scores. Similar findings are reported in recent cohort studies by La et al. and Luna et al. [22, 23].

The primary aim of our study was to assess patient outcomes via an English-lanugage translation of the QoLSPP questionnaire for those who underwent PP insertion post-ischaemic priapism in our institution, a quaternary referral centre between the years of 2002–2022. Our secondary aim was to record further qualitative data that was not captured within the QoLSPP but might aid the consent process for this procedure in the future.

## Methods

A retrospective cohort of patient’s having undergone PP insertion after an ischaemic priapism episode at University College London Hospital, a large Urological quaternary referral centre based in London, United Kingdom, was established from the hospital’s electronic archive for the period 2002–2022. Procedures were performed by a number of sub-specialised surgeons and devices utilised included both the IPP and MPP subtypes. All patients, except those recorded as deceased, were included in the study. Informed consent was obtained from all survey respondents. In discussion with the local ethics board (UCLH ethics board) and utilising the decision algorithm of the national ethics board in the UK (Health Research Authority) the study met criteria for exemption for formal research ethic committee approval.

An attempt to contact included patients was made by telephone, by 3 of the authors (AL, ID, FH) during a 6-week time period from November to December 2022. Researchers provided a detailed explanation of the survey and informed consent was obtained. The survey was then undertaken at the time by telephone or by follow-up email, depending on the patient’s preference The majority of the survey consisted of the QoLSPP questionnaire. The QoLSPP includes 16 questions across 4 domains (functional, personal, relational, social). Patients answered each question on a Likert scale of 0–5 in relation to their general and sexuality QoL over the past month. A satisfactory score on the QoLSPP questionnaire is a score of 3 or higher. We chose to add two additional questions to our survey:

1. How do you feel about the idea of living the rest of your sexual life in this condition? (Answered on a Likert scale 0–5)
2. If you could go back to your initial presentation with priapism, would you still choose to have a PP inserted? (Answered Yes/No)

Finally, the respondents were asked if they had any anecdotal comments they wished to have recorded regarding their experience of PP insertion.

Demographic details were logged. Clinical data regarding underlying conditions were retrospectively collected, as was the type of operation carried out (e.g. MPP or inflatable PP insertion) and the patients’ post-operative courses.

Categorical variables are reported as frequency and percentage, and continuous variables as median and interquartile range (IQR) or mean and standard deviation (SD). Regarding the QoLSPP question items, the main hypothesis to be tested was whether a significant proportion of question scores were ≥3 for a given question (a satisfactory score on the QoLSPP questionnaire is a score of 3 or higher). The null hypothesis was therefore that the proportion of significant scores (≥3) would not be significant (e.g. equal to 0.5). Z-tests were used to compare the proportion of patients with a satisfactory score to the null hypothesis proportion of 0.5. In addition, unpaired T-tests were used to compare mean question item scores by prosthesis sub-type. Statistical analysis was performed using Microsoft ® Excel 2023. All reported p-values are two-sided, and statistical significance was determined at *p* < 0.05.

## Results

**Fig. 1:**
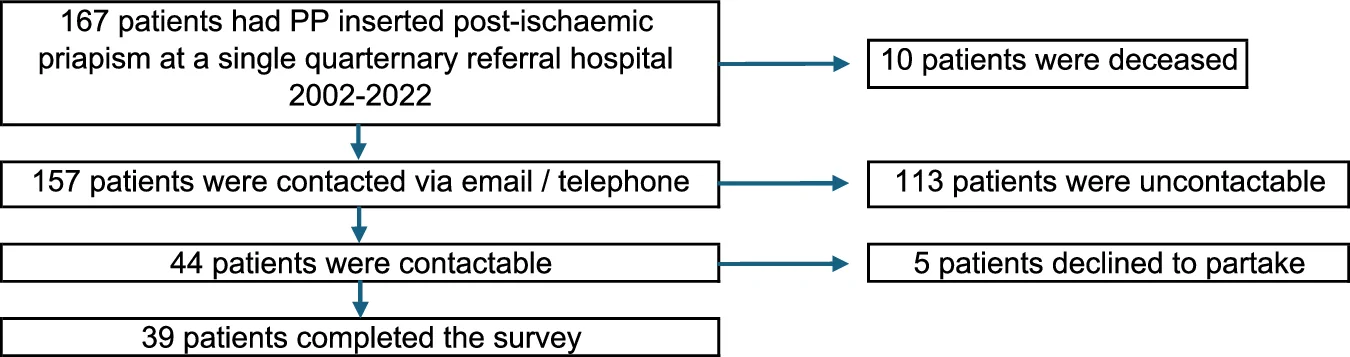
Flow chart of survey respondent recruitment. *PP* penile prosthesis.

In total, 167 patients underwent PP insertion in the post-ischaemic priapism setting between 2002 and 2022 at our institution. At the time of the survey, 10 of these patients were recorded as being deceased. Of the 157 remaining patients, 39 (24.8%) were contactable by phone and consented to completing the survey by phone or email (Fig. 1).

These 39 included patients formed our retrospective study cohort. The included patients had their devices inserted between 2007 and 2021. At the time of calling, 37 (94.9%) reported using their PP within the past month. The median duration post initial PP insertion to the study date was 9 years (IQR 3–11). The median age at the time of the survey was 56 years (IQR 50–63) and the median age at the time of PP insertion post-ischaemic priapism was 47 years (IQR 38–52). The median time from priapism episode to PP insertion was 15 days (IQR 7–49). Nine patients (23.1%) were recorded as having sickle-cell disease as the causative factor of priapism. The most commonly inserted erectile device in the initial setting was the MPP (25 patients, 64.1%) and at the time of the survey 17 patients (43.6%) had undergone revision surgery; 3 to replace MPP devices and 14 had an exchange from MPP to IPP devices. Demographics and clinical details are further outlined in Table 1.

**Table 1:** Baseline characteristics of the survey respondents.

Characteristic		Survery respondents (n = 39) median [IQR] / n (%)
Age at time of survey (years)		56 [50–63]
Age at time of initial erectile device insertion (years)		47 [38–52]
Interval from priapism episode to PP insertion (days)		15 [7–49]
Interval from time of priapism to survey (years)		9 [3–11]
Cause of priapism	Sickle Cell Disease	9 (23.1)
	Intracavernosal Alprostadil	3 (7.7)
	PDE5I	2 (5.1)
	Other medicines	4 (10.3)
	Other recorded reason (trauma, malignancy)	5 (12.8)
	Unknown	16 (41)
Initial device	Malleable	25 (64.1)
	Inflatable	14 (35.9)
Number of respondents who had undergone revision survey at time of survey		17 (43.6)
Revision type	MPP exchange	3 (7.7)
	MPP exchange to IPP	14 (35.9)

*IQR* interquartile range, *PP* penile prosthesis, *PDE5I* phosphodiesterase 5 inhibitor, *MPP* malleable penile prosthesis, *IPP* inflatable penile prosthesis.

**Fig. 2:**
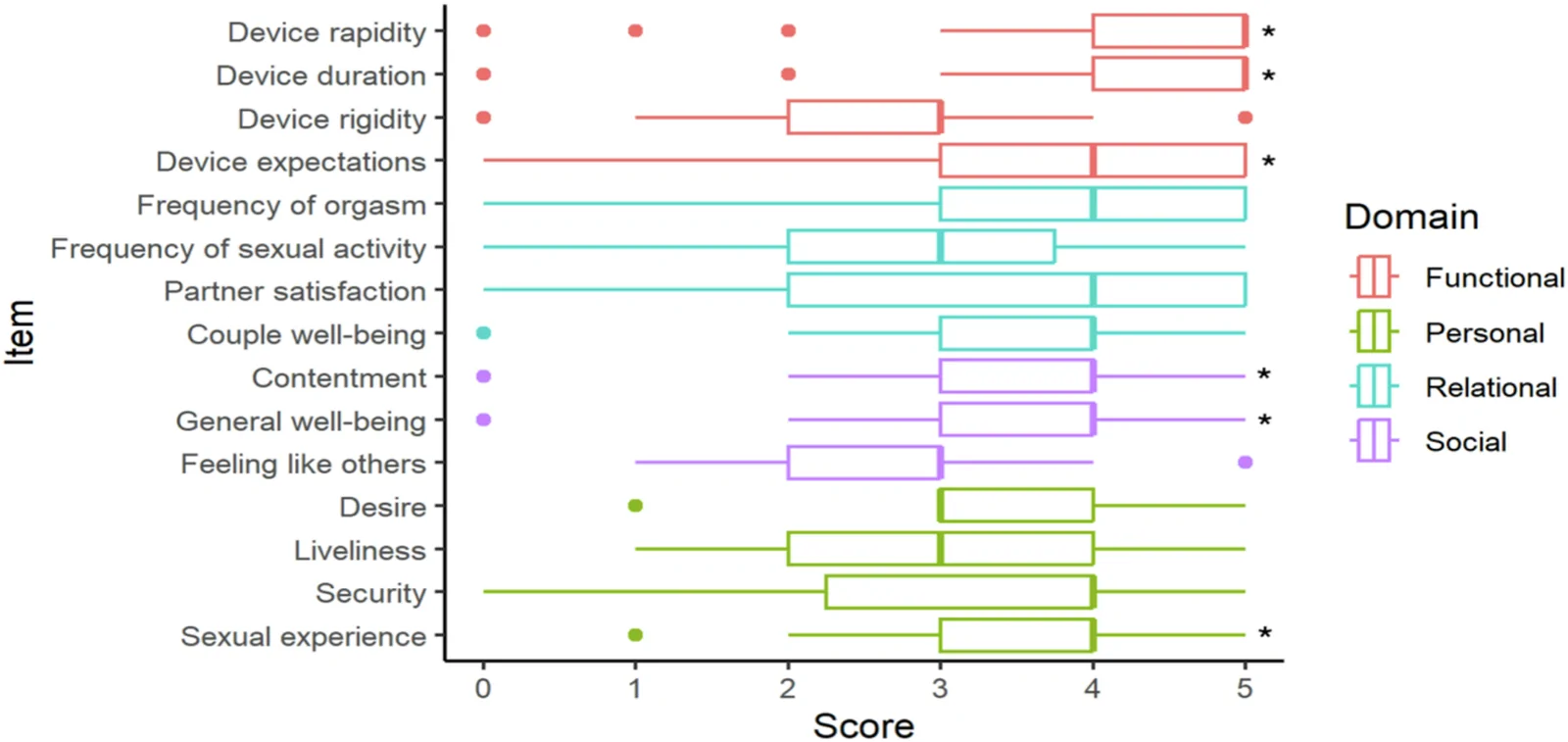
Box and whisker plot of the quality of life and sexuality with penile prosthesis item scores across 4 domains (functional, personal, relational and social). A two-tailed Z-test was used to determine which question items resulted in satisfactory scores (satisfactory scores Likert scale 3–5); ^*^denotes significant scores (*p* < 0.05).

Analysing mean item responses using Z-tests, the PP resulted in satisfactory scores (3, 4 or 5) at statistically significant (*p* < 0.05) levels in 7 of 16 items: device adequacy, device rapidity, device duration, meeting expectations, contentment with life, general well-bring and sexual experience. The highest level of significance (*p* < 0.001) was met by 3 items: device rapidity, duration and sexual experience. The mean score was not deemed at a statistically significant level of satisfaction for the remaining 9 items: device rigidity, frequency of orgasm, frequency of sexual activity, partner satisfaction, couple well-being, feeling like others, desire, liveliness, and security. Of note, the highest mean score was for device duration (4.3 ± 1.4) and the lowest was device rigidity (2.7 ± 1.1). The pooled mean score by domain was 3.7 ± 1.2 for functional, 3.3 ± 1.4 for relational, 3.2 ± 1 for social and 3.4 ± 1.1 for personal (Fig. 2 and Table 2).

**Table 2:** Quality of Life and Sexuality with Penile Prosthesis survey scores; items were answered on a Likert scale from 0–5 and the sum, mean and standard deviation (*SD*) calculated.

Domain	Item	Mean score (SD)	P-value
Functional	Device adequacy	3.5 (1.6)	0.002^*^
	Device rapidity	4.2 (1.5)	<0.001^*^
	Device duration of effects	4.3 (1.4)	<0.001^*^
	Device rigidity	2.7 (1.1)	0.423
	Device met expectations	3.8 (1.5)	0.007^*^
Relational	Frequency of orgasm	3.6 (1.5)	0.15
	Frequency of sexual activity	2.8 (1.4)	0.631
	Partner satisfaction	3.3 (1.6)	0.150
	Couple well-being	3.5 (1.1)	0.078
Social	Contentment with life	3.6 (1.1)	0.0163^*^
	General well-being	3.4 (1.0)	0.006^*^
	Feeling like others	2.8 (0.8)	0.150
Personal	Desire	3.5 (1.1)	0.423
	Liveliness	3.1 (1.0)	0.423
	Security	3.2 (1.5)	0.078
	Sexual experience	3.8 (0.9)	<0.001^*^

Pooled mean scores are given for each domain. A two-tailed Z-test was used to determine which question items resulted in satisfactory scores (satisfactory scores Likert scale 3–5) at statistically significant levels.

^*^denotes significant scores (*p* < 0.05).

Comparison of mean item scores for patients with IPP versus MPP sub-types demonstrated no statistically significant difference across all questions in the QoLSPP (Table 3). We discuss the potential limitation imposed by low patient numbers in our discussion.

**Table 3:** Quality of Life and Sexuality with Penile Prosthesis survey scores with comparison of mean and standard deviation by prosthesis sub-type (malleable vs inflatable).

		Mean score (SD)		
Domain	Item	IPP	MPP	P-value
Functional	Device adequacy	3.9 (1.4)	3 (1.7)	0.125
	Device rapidity	4.3 (1.1)	4.1 (2.1)	0.658
	Device duration of effects	4.5 (1.3)	4.2 (1.9)	0.584
	Device rigidity	3 (1.1)	2.9 (1.3)	0.746
	Device met expectations	4 (1.3)	4 (1.8)	1.000
Relational	Frequency of orgasm	3.2 (1.3)	2.9 (1.4)	0.486
	Frequency of sexual activity	3 (1.1)	2.9 (1.3)	0.746
	Partner satisfaction	3.6 (1.8)	2.9 (1.8)	0.342
	Couple well-being	3.7 (1.7)	3.2 (1.2)	0.384
Social	Contentment with life	3.8 (1.2)	3.5 (1)	0.412
	General well-being	3.5 (1.1)	3.2 (1)	0.479
	Feeling like others	2.9 (0.7)	2.8 (0.9)	0.650
Personal	Desire	4 (0.8)	3.6 (1.1)	0.285
	Liveliness	3.3 (0.9)	3.1 (1)	0.542
	Security	3.1 (1.7)	3.3 (1.4)	0.830
	Sexual experience	3.6 (1)	3.3 (0.9)	0.417

A two-tailed T-test was used to determine statistically significant differences (*p* < 0.05). (There were no statistically significant differences).

*IPP* inflatable penile prosthesis, *MPP* malleable penile prosthesis.

Regarding the additional questions posed to respondents:

1. All 39 respondents (100%) agreed that if they were to return to their initial presentation with priapism, they would still choose PP insertion as their treatment option.
2. All 39 respondents (100%) would be satisfied (Likert score 3,4, or 5) living the remainder of their lives with a PP in situ (134/195, Mean score 3.4).

Finally, the respondents were asked if they had any other comments they wished to have recorded regarding their experience of PP insertion. Ten respondents cited specific reasons as the cause of not being satisfied with their PP – these can be seen in Table 4 below.

**Table 4:** Summary table of anecdotal causes of patient dissatisfaction with penile prostheses.

Cause for patient dissatisfaction	n (%)
Reduced penile length	2 (5.1%)
Reduced penile length and girth	3 (7.7%)
Preference for IPP placement in initial setting	2 (5.1%)
‘Abnormal’ penis shape	1 (2.6%)
Negative impact of informing new sexual partners	1 (2.6%)
Partner reporting reduced sensation	1 (2.6%)

## Discussion

Our work is unique as we evaluate the experience of PP patients with end-stage ED due to ischemic priapism using the validated QoLSPP questionnaire. Ischemic priapism represent a minority of the PP cohort, therefore there is little published data on patient reported outcomes in this group. We believe this to be the largest study to date assessing QoL outcomes for PP post-ischaemic priapism.

Amongst our cohort of 39 patients, the PP resulted in satisfactory scores in 7 of 16 items: device adequacy, device rapidity, device duration, meeting expectations, contentment with life, general well-being and sexual experience. Satisfactory scores for device adequacy, rapidity and duration suggests PP device mechanism and operability are currently meeting adequate standards for use in sexual intercourse. This is particularly reassuring for the device given the median time from insertion to questionnaire was 9 years. Satisfactory scores in relation to general contentment with life, well-being and sexual experience are positive findings for patient’s overall feelings. These are broad questions which suggest a general satisfaction with life and sexuality with a PP.

The PP did not reach satisfactory scores in the remaining 9 of 16 questions: device rigidity, frequency of orgasm, frequency of sexual activity, partner satisfaction, couple well-being, feeling like others, desire, liveliness, and security. Lack of satisfactory scores in frequency of orgasm and sexual activity is likely to relate to a small proportion of the cohort (5.1%) whom were no longer sexually active or had exited relationships since their device insertion. These patients therefore answered 0 in relation to frequency of orgasm and sexual activity. Lack of satisfactory scores in couple well-being and partner satisfaction may imply difficulties related to partner experience and feelings towards PP. Sexual satisfaction of the patient is largely influenced by their partner’s satisfaction, which makes partner satisfaction rates an important parameter [24]. It would be of interest to explore this further through partner-reported outcomes in future work. Lack of satisfactory scores related to security and rigidity raises concerns that patients may not feel safe using their prosthesis or that it reaches inadequate hardness for penetration. ‘Liveliness’ we identified to be a question most patients found interpret in the English language translation of the QoLSPP. Therefore lack of satisfaction in this question we deem not to be of significance.

The domain with the highest pooled mean score was 3.7 ± 1.2 for functional. This domain also included the highest mean score which was for device duration (4.3 ± 1.4). Again, this is suggestive that device mechanisms are adequate as patients are satisfied with duration for sexual intercourse. In regards to the other three domains: we found pooled scores of 3.3 ± 1.4 for relational, 3.2 ± 1 for social and 3.4 ± 1.1 for personal. All of these pooled domain scores are above the level of 3, deemed a satisfactory score.

Regarding our additional questions on sexual QoL, none of the 39 patients regretted the decision to have a PP placed and all felt satisfied living with the prosthesis for the duration of their lives. Whilst non-validated questions, these responses are very reassuring for the impact PP insertion has for ischaemic priapism patients. That, regardless of not meeting all expectations, patients are still overall happy to have made a decision for surgery. This finding can be integrated into how clinicians should seek to counsel post-ischaemic priapism patients for PP pre-operatively.

There was failure to find statistical significance comparing prosthesis sub-type. This is significant and may indicate a genuine lack of difference in significance of PP type. However, satisfaction rates have previously been observed to be more favourable amongst IPP patients compared to MPP [12–16]. Our failure to identify significance we believe it is more likely to indicate insufficient power in the study and should be interpreted with caution.

Amongst the available literature, there has been a heterogenous approach to assessing sexual QoL outcomes amongst PP patients. One of the first studies to address psychosocial follow up from PP implantation conducted by Tiefer et al. in 1988 emphasises the methodological difficulties in measuring satisfaction following PP insertion [25]. These authors utilised structured interviews and classified anecdotes as positive or negative comments. A more recent observational study by Zacharakis et al. compared early versus delayed PP implantation in a cohort of 95 patients with post-ischaemic priapism ED. They demonstrate 96% patient satisfaction in the early implantation group compared to 60% in the delayed implantation group. However, in that cohort, patient satisfaction was measured solely on the basis of completion of sexual intercourse [14]. Other notable studies have adopted this simplistic approach, basing QoL ‘satisfaction’ on achievement of sexual intercourse [13, 14, 26].

Most of the available evidence on patient satisfaction after PP insertion is unreliable due to the use of questionnaires validated in different patient settings. Kılıçarslan et al. address QoL outcomes for patients with PP utilising a modified version of the Erectile Dysfunction Inventory of Treatment Satisfaction score (EDITS) [5]. They find IPP as more successful in overall satisfaction compared to the MPP amongst 46 patients. Other questionnaires utilised include the Index of Male Genital Image (IMGI), and the International Index of Erectile Function score (IIEF) [27]. None of these questionnaires have been designed specifically for evaluation of the ED patient who has had a PP inserted. The use of a questionnaire validated previously in a non-PP population could lead to an improper estimation of patient satisfaction for a PP cohort. This is because, measures such as EDITS, IMGI and IIEF lack the evaluation of relevant aspects unique to PP such as device operability, rigidity and function.

We chose to utilise the QoLSPP in this work as it has been applied and validated repeatedly. Manfredi et al. have recently pooled data from 8 available studies reporting on QoLSPP outcomes [28]. However, a key issue remains, that the QoLSPP questionnaire was designed in Italian. Although it has previously been applied as an English language version multiple times, these studies consistently report it has terminology that can be difficult to interpret for English patients [21–23]. More recently, Salter et al. have developed the Satisfaction Survey for Inflatable Penile Implant (SSIPI) as a validated English-language questionnaire to assess patient satisfaction after IPP surgery [29]. It would be interesting to see future studies compare outcomes in a single PP cohort measured by an English language QoLSPP versus SSIPI.

Manfredi et al. report on a systematic review of QoLSPP scores across PP studies and present pooled scores based on 693 patients across 8 published studies [28]. Their analysis demonstrates pooled scores of 4.22 (95% CI 4.04–4.40), 4.17 (95% CI 4.03–4.31), 4.21 (95% CI 3.61–4.32) and 3.97 (95% CI 3.61–4.32) in the functional, relational, social and personal domains respectively. In comparison to our post-ischaemic priapism cohort, these authors present overall higher QoLSPP scores across all domains for the PP cohort as a whole. This may be due to the previously discussed challenges in achieving full satisfaction with PP post-ischaemic priapism due to tissue damage resulting in potential penile deformity, shortening or limited PP size [30]. Indeed, the anecdotal data we collected on causes of dissatisfaction included concerns of reduction in penile length, girth and abnormal penis shape. Further, This serves to emphasise the importance of counselling and expectation setting for insertion of a PP for ischaemic priapism patients.

Patients with priapism face an increased risk of infection, erosion and contracture leading to increased revision rates [31]. Within our cohort, 17 patients (43.6%) had undergone revision surgery. This is substantially higher than the overall re-operation rate of 7.42% amongst the total penile prosthesis population [32]. Patients undergoing re-operation are less likely to be satisfied as they may feel their surgery has failed to meet expectations, they are more likely to have experience side-effects leading to re-operation and are likely to present more challenging cases in their presentation. This is an additional important factor which may contribute to lower satisfaction amongst the post-ischaemic priapism PP cohort.

The uncommon incidence of PP insertion post-ischaemic priapism is a key limiting factor in being able to survey larger patient cohorts. The significant attrition of patients at follow-up for post-ischaemic priapism likely contributes to the lower PP insertion rates in this group. It is worth considering that post-ischaemic priapism patients represent a challenging group in adherence to follow up. In particular, amongst sickle cell patients (representing the main aetiology of 23% of adult cases of priapism), priapism is associated with a high rate of unrecognised mental health challenges, including suicidality, depression and anxiety [33, 34]. These factors could preclude patients from seeking medical follow-up. This low retention of patients post-ischaemic priapism represents an inherent limitation of both previous studies of post-ischaemic priapism PP outcomes as well as our own.

Our study had several other limitations that we have yet to acknowledge. The QoLSPP questionnaire has inherent limitations beyond just it’s language barrier. The absence of partner-reported outcomes weakens the relational aspect of the questionnaire, as sexual satisfaction often depends on mutual satisfaction. The small study population (39 respondents) is understandable given the rarity of priapism, but limits the generalisability of our findings. In addition, the low response rate to the survey (24.8%) raises concern that there may have been selection bias introduced into the study population. Those who declined to partake due to dissatisfaction, or those lost to follow-up, might differ systematically from respondents. Further limitation includes the lack of control group in this study (e.g. post-priapism patients without PP), making it difficult to fully contextualise the QoLSPP scores as attributable to the PP.

In this study, we did not compare outcomes for patients with delayed versus early implantation of PP post-ischaemic priapism. We would highlight this limitation as particularly significance given that a greater understanding of whether delaying PP insertion after a priapism episode could potentially alleviate the psychological impact of the surgery. Such a delay may afford patients the necessary time to gain a better understanding of their condition and afford greater QoL outcomes with device use lifelong. A recently published comprehensive review by Abou Chawareb et al. has concluded the superiority of early implantation over delayed implantation for patients who have experienced ischaemic priapism [35]. However, this field requires further study to understand the significance for patient QoL of an early versus delayed surgery.

## Conclusions

In conclusion, we report on QoL outcomes of patients receiving PP post-ischaemic priapism, utilizing an English language translation of the validated QoLSPP. Findings show significant satisfaction scores in the following 7 of 16 QoLSPP items: device adequacy, device rapidity, device duration, meeting expectations, contentment with life, general well-being and sexual experience. None of the patients regretted the decision to insertion and all felt satisfied having it for the remainder of their lives. Our results should be interpreted with caution. There is a lack of robust evidence on this topic, both previously, and reported here due to the rarity of the condition. Insertion of a PP represents a unique opportunity for a small cohort of males who have experienced ischemic priapism with subsequent end-stage refractory ED to achieve restorative function of the penis. Now, a validated English language questionnaire should be applied in a larger cohort for stronger results. The SSIPI represents a reasonable alternative. Dissatisfaction can be addressed through preoperative counselling and building realistic post-operative expectations particularly regarding dissatisfaction with penile length and rigidity. This requires shared decision-making between the patient and surgeon, including a detailed discussion about the risks that are unique to PP surgery in the setting of post-ischaemic priapism. Patients awaiting PP post-ischaemic priapism often have less time for decision making and this may also impact our ability to counsel patients fully, in order to set honest expectations. We hope the evidence from this data will help in assisting patients with post-ischaemic priapism surgical options.

## Data Availability

Data generated or analysed during this study can be found within the published article.
